# On the Wave Turbulence Theory for the Nonlinear Schrödinger Equation with Random Potentials

**DOI:** 10.3390/e21090823

**Published:** 2019-08-23

**Authors:** Sergey Nazarenko, Avy Soffer, Minh-Binh Tran

**Affiliations:** 1 Institut de Physique de Nice, Université Côte d’Azur, Centre national de la recherche scientifique (CNRS), Parc Valrose, 06108 Nice, France; 2 Mathematics Department, Rutgers University, New Brunswick, NJ 08903 USA; 3 Department of Mathematics, Southern Methodist University, Dallas, TX 75275, USA

**Keywords:** wave turbulence theory, nonlinear schrödinger equation with random potentials, 4-wave kinetic turbulence equation, ohm’s law, porous medium equation.

## Abstract

We derive new kinetic and a porous medium equations from the nonlinear Schrödinger equation with random potentials. The kinetic equation has a very similar form compared to the four-wave turbulence kinetic equation in the wave turbulence theory. Moreover, we construct a class of self-similar solutions for the porous medium equation. These solutions spread with time, and this fact answers the “weak turbulence” question for the nonlinear Schrödinger equation with random potentials. We also derive Ohm’s law for the porous medium equation.

## 1. Introduction

The nonlinear Schrödinger equation (NLSE) with random potentials is a fundamental problem in both mathematical and physical research. Although there have been extensive mathematically rigorous, analytical and numerical results, several elementary properties of the dynamics of the solutions are still not known. The resolution of the problem plays a central role in understanding several physical phenomena in chaos and nonlinear physics. The NLSE with random potentials is written as follows,

(1) $$\begin{array}{cc} i\partial_{t}\Psi \left(x,t\right)\;= & \;H_{0}\Psi \left(x,t\right)\;+\;\epsilon {\left|\Psi \left(x,t\right)\right|}^{2}\Psi \left(x,t\right)\\ = & \;-\Delta \Psi \left(x,t\right)\;+\;V_{x}\Psi \left(x,t\right)\;+\;\epsilon {\left|\Psi \left(x,t\right)\right|}^{2}\Psi \left(x,t\right), \end{array}$$

where $V_{x}$ is a random function and ϵ is a constant.

On a one-dimensional lattice,

x∈ξZ:={ξn,n∈Z},

the lattice version of the above equation can be written as

(2) $$\begin{array}{cc} i\partial_{t}\Psi_{x}\;= & \;\frac{1}{\xi^{2}}\left[\Psi_{x+\xi }+\Psi_{x-\xi }-2\Psi_{x}\right]\;+\;\epsilon {\left|\Psi_{x}\right|}^{2}\Psi_{x}+\;V_{x}\Psi_{x}, \end{array}$$

where $V_{x}$ is a collection of i.d.d. random variables uniformly distributed in the interval [−ω/2,ω/2]. In this paper, we will focus on the so-called “weak turbulence” question about the dynamics of the solution in large time:

(3) $$\begin{array}{c} \mathrm{Will}a\mathrm{small}\mathrm{nonlinearity}\mathrm{spread}\mathrm{the}\mathrm{solution}\mathrm{over}\mathrm{distances}\mathrm{much}\mathrm{greater}\mathrm{than}\\ \mathrm{the}\mathrm{linear}\mathrm{system}\mathrm{does}\mathrm{for}\mathrm{large}\mathrm{times}\mathrm{for}\mathrm{an}\mathrm{initial}\mathrm{condition}\mathrm{localized}\\ \mathrm{in}\mathrm{space}\mathrm{and}\mathrm{frequency}? \end{array}$$

This question is still open despite several efforts [1,2,3,4,5,6,7,8,9,10,11]. The resolution of this question may shed lights on many nonlinear problems, such as the famous Fermi–Pasta–Ulam–Tsingou (FPUT) problem [12,13]. We also refer to [14,15,16] for recent numerical works on the microcanonical Gross–Pitaevskii (also known as the semiclassical Bose–Hubbard) lattice model dynamics.

A convincing evidence for delocalisation by nonlinearity has been gained by numerical experiments, and several intuitive arguments and phenomenological descriptions were suggested. However, there still remains a need for a more solid theory based on more rigorous and systematic derivations with clear starting assumptions. In this work, we introduce a new class of models that can be analyzed by wave turbulence theory. There are new features to this model, namely, the built-in randomness. Since the assumption of random phase is a crucial one in deriving the kinetic equation, this model offers a new way of verifying this step. To be more precise, our approach is to use the wave turbulence approach [17,18,19,20,21] to derive a kinetic equation from the 1D lattice NLSE with random potentials (1). Superficially, the derived kinetic equation has a similar form as the four-wave turbulence kinetic equation and the quantum Boltzmann equation [20,22,23,24,25,26,27,28]. However, there is no conservation of momentum due to the localisation in space of the linear modes, and the modes are parametrised by their location on the lattice rather by their momentum–space location. On the other hand, it is the localised nature of the linear modes and the fact that the interactions happen only locally in physical space that allow us, in the first order approximation, to transform the kinetic equation into a nonlinear diffusion equation, a.k.a. the porous medium equation. For the porous medium equation, we construct a self-similar solution that spreads for large times. This fact answers positively the “weak turbulence” question (3). We also find a class of steady state solutions to the equation, that lead to a new nonlinear Ohm’s law. This result implies that small nonlinearity breaks down the insulator property of the lattice and turns it into a nonlinear conductor. Despite the numerical evidence for chaotic behavior of the NLS with random potential, and of similar fundamental systems, like FPUT, randomized Klein Gordon type equations and more, there is no compelling analytic or even phenomenological explanation. So far, all rigorous results only point to long time delocalization. Therefore, this work offers a new, analytic way, with explicit assumptions, to understand the spreading phenomena. Let us point out that we do not claim that the assumptions we made are easily proven. Quite the contrary, complete rigorous proof of this result would be a transformative piece of work in mathematics and chaos theory. The assumptions we listed can be divided into two classes: one needed to derive the kinetic equation and the other one needed to approximate the behavior of the kinetic equation by a partial differential equation. The crucial part is the first class of assumptions. The second class can be verified by numerical simulations of the kinetic equations on the relevant time scale. This is indeed a huge simplification, since observing the spreading numerically requires solving the NLS on a very large time scale ($10^{6}$–$10^{11}$), and it is not clear if the numerics really follows the solution of the equation. The first class of assumptions are mainly of the same type, that the *n*-point functions, averaged on the random potential become random-phased quasi-free. Some of them are in fact conditions on the linear Anderson model and, therefore, amenable to rigorous analysis. Presence of random potential makes our problem essentially different from a standard wave turbulence system in which the only source of randomness is in the initial data. Thus, in standard wave turbulence, the initial randomness is assumed to be propagating over the nonlinear time, whereas in presence of random potentials, it is naturally present at all times. We, therefore, believe our work lays the basis to a new way of studying the above problem, with specific road map assumptions of general interest.

## 2. Derivation of the Kinetic Equation

There have been several approaches to deriving wave turbulence in the previous literature. For our purposes, the most suited, is the wave turbulence technique of [20] (see also the original papers [19,29,30,31]). This approach is based on an explicit formulation of statistical properties of waves by introducing ”the random phase and amplitude" fields. However, due to the localisation of the linear modes in presence of random potential, we will have to adopt an extra element previously developed in [32], assuming Wick’s type behaviour of Nth order correlations of the linear problem; the so-called quasi-free field assumption.

### 2.1. Dynamical Equations for the Mode Amplitudes

Let us represent the the wavefunction in terms of the eigenvalues $E_{j}$, computed in [32], and eigenvectors $\psi_{j}\left(x\right)$ of the Hamiltonian $H_{0}$:

$$\Psi \left(x,t\right)\;=\;\sum_{j\in Z}c_{j}\left(t\right)e^{-iE_{j}t}\psi_{j}\left(x\right).$$

Substituting the above expansion into (2) and removing all the oscillating linear terms, we find

(4) $$\partial_{t}c_{j}\left(t\right)\;=\;\epsilon \sum_{l,m,n\in Z}V_{jl}^{mn}c_{l}^{*}c_{m}c_{n}e^{i(E_{n}-E_{l}+E_{m}-E_{j})t}\;=:\;\epsilon \sum_{l,m,n\in Z}V_{jl}^{mn}c_{l}^{*}c_{m}c_{n}e^{iE_{lj}^{mn}t},$$

in which

(5) $$V_{jl}^{mn}\;=\;\sum_{x\in \xi Z}\psi_{l}^{*}\left(x\right)\psi_{m}\left(x\right)\psi_{n}\left(x\right)\psi_{j}^{*}\left(x\right)=V_{mn}^{jl*}.$$

For further derivation, we need to remove the diagonal terms, which include terms satisfying (n,m)=(l,j) and (n,m)=(j,l). These terms can be expressed as follows,

$$\sum_{\left(n,m)=(l,j),(n,m)=(j,l\right)}V_{jl}^{mn}c_{l}^{*}c_{m}c_{n}e^{i(E_{n}-E_{l}+E_{m}-E_{j})t}\;=\;2c_{j}\sum_{n\in Z}V_{jn}^{jn}{\left|c_{n}\right|}^{2}\;=:\;E_{NL}c_{j}.$$

We then absorb these terms by defining the energy renormalization

(6) $$E\;:=\;E\;+E_{NL},$$

that gives

(7) $$\partial_{t}c_{j}\left(t\right)\;=\;\epsilon \sum_{l,m,n\in Z}^{\prime }V_{jl}^{mn}c_{l}^{*}c_{m}c_{n}e^{i(E_{n}-E_{l}+E_{m}-E_{j})t}=:\;\epsilon \sum_{l,m,n\in Z}^{\prime }V_{jl}^{mn}c_{l}^{*}c_{m}c_{n}e^{iE_{mn}^{jl}t},$$

where $\sum_{l,m,n\in Z}^{\prime }$ denotes the sum in which the diagonal terms (n,m)=(l,j) and (n,m)=(j,l) are excluded.

So far we have not made any approximations and our ODE system (7) is equivalent to the original equation (2).

### 2.2. Weak Nonlinearity Expansion

Let us now introduce the intermediate time *T*

(8) $$\frac{2\pi }{E_{j}}\ll \;T\ll \;\frac{2\pi }{E_{j}\epsilon^{2}}.$$

For T in this range our approximations will make sense for most potentials (probability close to 1). Under the assumption that ϵ is very small, we can expand the coefficient $c_{j}\left(T\right)$ as

(9) $$c_{j}\left(T\right)\;=\;c_{j}^{\left(0\right)}\left(T\right)\;+\;\epsilon c_{j}^{\left(1\right)}\left(T\right)\;+\;\epsilon^{2}c_{j}^{\left(2\right)}\left(T\right)\;+\;\cdots$$

Inserting the expansion (9) into (7), yields a new system of equation for $c_{j}^{\left(r\right)}$ that we describe below.

For r=0, the problem is linear, and we have the following equation

(10) $$\partial_{t}c_{j}^{\left(0\right)}\;=\;0,$$

that implies

(11) $$c_{j}^{\left(0\right)}\left(T\right)\;=\;c_{j}^{\left(0\right)}\left(0\right).$$

For r=1, we find

(12) $$i\partial_{t}c_{j}^{\left(1\right)}\;=\;\sum_{m,n,l\in Z}^{\prime }V_{lj}^{mn}e^{iE_{lj}^{mn}t}c_{m}^{\left(0\right)}c_{n}^{\left(0\right)}c_{l}^{(0)*}$$

which yields

(13) $$c_{j}^{\left(1\right)}\;=\;-i\sum_{m,n,l\in Z}^{\prime }V_{lj}^{mn}\Delta_{T}\left(E_{lj}^{mn}\right)c_{m}^{\left(0\right)}c_{n}^{\left(0\right)}c_{l}^{(0)*},$$

where

$$\Delta_{T}\left(E_{lj}^{mn}\right)\;=\;\int_{0}^{T}e^{iE_{lj}^{mn}t}dt\;=\;\frac{e^{iE_{lj}^{mn}T}-1}{iE_{lj}^{mn}}.$$

For r=2, the following equation can be obtained,

(14) $$\begin{array}{cc} i\partial_{t}c_{j}^{\left(2\right)}\;= & \;\sum_{m,n,l\in Z}^{\prime }V_{lj}^{mn}e^{iE_{lj}^{mn}t}\left(2c_{m}^{\left(1\right)}c_{n}^{\left(0\right)}c_{l}^{(0)*}+c_{m}^{\left(0\right)}c_{n}^{\left(0\right)}c_{l}^{(1)*}\right)\\ \;= & \;\;-i\sum_{m,n,l\in Z}^{\prime }V_{lj}^{mn}e^{iE_{lj}^{mn}t}(2c_{n}^{\left(0\right)}c_{l}^{(0)*}\sum_{\lambda ,\mu ,\nu \in Z}V_{\lambda m}^{\mu \nu }\Delta_{T}\left(E_{\lambda m}^{\mu \nu }\right)c_{\mu }^{\left(0\right)}c_{\nu }^{\left(0\right)}c_{\lambda }^{(0)*}\\ & \;-c_{m}^{\left(0\right)}c_{n}^{\left(0\right)}\sum_{\lambda ,\mu ,\nu \in Z}^{\prime }V_{\lambda l}^{\mu \nu *}\Delta_{T}^{*}\left(E_{\lambda l}^{\mu \nu }\right)c_{\mu }^{(0)*}c_{\nu }^{(0)*}c_{\lambda }^{\left(0\right)}), \end{array}$$

which yields

(15) $$\begin{array}{cc} c_{j}^{\left(2\right)}\;= & \;\;-\sum_{m,n,l,\lambda ,\mu ,\nu \in Z}^{\prime }V_{lj}^{mn}[2c_{n}^{\left(0\right)}c_{l}^{(0)*}c_{\mu }^{\left(0\right)}c_{\nu }^{\left(0\right)}c_{\lambda }^{(0)*}V_{\lambda m}^{\mu \nu }\Gamma \left(E_{lj}^{mn},E_{\lambda m}^{\mu \nu }\right)\\ & \;-c_{m}^{\left(0\right)}c_{n}^{\left(0\right)}c_{\mu }^{(0)*}c_{\nu }^{(0)*}c_{\lambda }^{\left(0\right)}V_{\lambda l}^{\mu \nu *}\Gamma \left(E_{lj}^{mn},E_{\mu \nu }^{\lambda l}\right)], \end{array}$$

where

$$\Gamma \left(x,y\right)\;=\;\int_{0}^{T}e^{ixt}\Delta_{t}\left(y\right)dt.$$

Now, let us try to understand the spectrum by developing

(16) $$\begin{array}{cc} N_{j}\left(T\right)\;= & \;\langle |c_{j}\left(T\right){|}^{2}\rangle \\ \;= & \;\langle |c_{j}^{\left(0\right)}\left(T\right)\;+\;\epsilon c_{j}^{\left(1\right)}\left(T\right)\;+\;\epsilon^{2}c_{j}^{\left(2\right)}\left(T\right)\;+\cdots {|}^{2}\rangle \\ \;= & \;\langle |c_{j}^{\left(0\right)}{\left(T\right)|}^{2}\rangle \;+\;\epsilon \langle c_{j}^{\left(1\right)}\left(T\right)c_{j}^{(0)*}\left(T\right)\;+\;c.c.\rangle \;+\;\epsilon^{2}\langle |c_{j}^{\left(1\right)}\left(T\right){|}^{2}\rangle \\ \; & \;+\;\epsilon^{2}\langle c_{j}^{\left(2\right)}\left(T\right)c_{j}^{(0)*}\left(T\right)\;+\;c.c.\rangle . \end{array}$$

### 2.3. Statistical Averaging

Fundamentally, stochasticity of our system arises from randomness of the potentials at each lattice site. Even if the initial mode amplitudes $c_{j}$ are deterministic, they will become random at a later time. Moreover, it is natural to assume that $c_{j}$’s will become statistically independent at each site *j* and that their phases will become random.

Thus, let us assume that, at beginning, such randomness is already ensured by the preceding evolution at t<0. Specifically, let us make the following assumptions:

- Assumption 1: Phase randomness. This is a standard wave turbulence assumption. We assume that the phases of $c_{j}^{\left(0\right)}$ are random and, therefore, we can use Wick’s pairing, which says that non-zero contributions only arise in paring $c_{j}^{\left(0\right)}$ and $c_{j}^{(0)*}$. That means the first order term in ϵ is 0
  $$\langle c_{j}^{(0)*}c_{j}^{\left(1\right)}\rangle \;=\;-i\sum_{m,n,l\in Z}^{\prime }\langle V_{lj}^{mn}c_{m}^{\left(0\right)}c_{n}^{\left(0\right)}c_{l}^{(0)*}c_{j}^{(0)*}\Delta \left(E_{ij}^{mn}\right)\rangle =0.$$
  This result holds because the diagonal terms with (m,l)=(n,j) and (m,n)=(l,j) are excluded from the sum.
  The second order terms in ϵ can be written as
  (17) $$\begin{array}{cc} \langle |c_{j}^{\left(1\right)}{|}^{2}\rangle = & \sum_{l,m,n,\lambda ,\mu ,\nu \in Z}^{\prime }\langle V_{lj}^{mn}\Delta_{T}\left(E_{lj}^{mn}\right)c_{m}^{\left(0\right)}c_{n}^{\left(0\right)}c_{l}^{(0)*}V_{\lambda j}^{\mu \nu *}\Delta_{T}^{*}\left(E_{\lambda j}^{\mu \nu }\right)c_{\mu }^{(0)*}c_{\nu }^{(0)*}c_{\lambda }^{\left(0\right)}\rangle \;\\ = & 2\;\sum_{m,n,l\in Z}^{\prime }\langle |V_{lj}^{mn}{|}^{2}|c_{m}^{\left(0\right)}{|}^{2}|c_{n}^{\left(0\right)}{|}^{2}|c_{l}^{\left(0\right)}{|}^{2}|\Delta \left(E_{lj}^{mn}\right){|}^{2}\rangle \end{array}$$
  and
  (18) $$\begin{array}{cc} \langle c_{j}^{\left(2\right)}c_{j}^{(0)*}\rangle \;= & \;-\sum_{m,n,l,\lambda ,\mu ,\nu \in Z}^{\prime }\langle V_{lj}^{mn}[2c_{n}^{\left(0\right)}c_{\mu }^{\left(0\right)}c_{\nu }^{\left(0\right)}c_{l}^{(0)*}c_{\lambda }^{(0)*}c_{j}^{(0)*}V_{\lambda m}^{\mu \nu }\Gamma \left(E_{lj}^{mn},E_{\lambda m}^{\mu \nu }\right)\\ & \;-c_{m}^{\left(0\right)}c_{n}^{\left(0\right)}c_{\lambda }^{\left(0\right)}c_{\mu }^{(0)*}c_{\nu }^{(0)*}c_{j}^{(0)*}V_{\lambda l}^{\mu \nu *}\Gamma \left(E_{lj}^{mn},E_{\mu \nu }^{\lambda l}\right)]\rangle \\ \;= & \;-2\sum_{m,n,l\in Z}^{\prime }\langle |V_{lj}^{mn}{|}^{2}\Gamma \left(E_{lj}^{mn},E_{mn}^{lj}\right)\left[2|c_{l}^{\left(0\right)}{|}^{2}|c_{n}^{\left(0\right)}{|}^{2}|c_{j}^{\left(0\right)}{|}^{2}-|c_{m}^{\left(0\right)}{|}^{2}|c_{n}^{\left(0\right)}{|}^{2}{\left|c_{j}^{\left(0\right)}\right|}^{2}\right]\rangle . \end{array}$$
- Assumption 2: Amplitude averaging, “quasi-free field” assumption. Here, we will assume that the mode amplitudes at each site are statistically independent. Moreover, we will assume that these amplitudes are independent from $|V_{lj}^{mn}{|}^{2}{\left|\Delta \left(E_{lj}^{mn}\right)\right|}^{2}$ and $|V_{lj}^{mn}{|}^{2}\Gamma \left(E_{lj}^{mn},E_{mn}^{lj}\right)$ (complicated functions of the random potentials which are fixed for each realisation). This assumption leads to
  (19) $$\begin{array}{cc} & \langle |V_{lj}^{mn}{|}^{2}|c_{m}^{\left(0\right)}{|}^{2}|c_{n}^{\left(0\right)}{|}^{2}|c_{l}^{\left(0\right)}{|}^{2}|\Delta \left(E_{lj}^{mn}\right){|}^{2}\rangle \\ =\; & \;\langle |V_{lj}^{mn}{|}^{2}|\Delta \left(E_{lj}^{mn}\right){|}^{2}\rangle \langle c_{m}^{\left(0\right)}{|}^{2}\rangle \langle |c_{n}^{\left(0\right)}{|}^{2}\rangle \langle |c_{l}^{\left(0\right)}{|}^{2}\rangle \end{array}$$
  and
  (20) $$\begin{array}{c} \langle |V_{lj}^{mn}{|}^{2}\Gamma \left(E_{lj}^{mn},E_{mn}^{lj}\right)\left[2|c_{l}^{\left(0\right)}{|}^{2}|c_{n}^{\left(0\right)}{|}^{2}|c_{j}^{\left(0\right)}{|}^{2}-|c_{m}^{\left(0\right)}{|}^{2}|c_{n}^{\left(0\right)}{|}^{2}{\left|c_{j}^{\left(0\right)}\right|}^{2}\right]\rangle =\\ \langle |V_{lj}^{mn}{|}^{2}\Gamma \left(E_{lj}^{mn},E_{mn}^{lj}\right)\rangle \left[2\langle |c_{l}^{\left(0\right)}{|}^{2}\rangle \langle |c_{n}^{\left(0\right)}{|}^{2}\rangle \langle |c_{j}^{\left(0\right)}{|}^{2}\rangle -\langle |c_{m}^{\left(0\right)}{|}^{2}\rangle \langle |c_{n}^{\left(0\right)}{|}^{2}\rangle \langle |c_{j}^{\left(0\right)}{|}^{2}\rangle \right] \end{array}$$

Note that the assumption about random statistically independent wave amplitudes is one of the key elements of the wave turbulence technique of [20]. However, the quasi-free field assumption goes beyond this assumption by additionally assuming that the wave amplitudes get decorrelated from $V_{lj}^{mn}$ and $E_{lj}^{mn}$ which contains the original randomness source via the random nature of the linear eigenmodes and eigenvalues.

### 2.4. Four-Wave Kinetic Equation

According to the inequality (8), the small nonlinearity limit corresponds to T→∞, and in this case we know that

(21) $$|\Delta \left(E_{lj}^{mn}\right){|}^{2}\to 2\pi T\delta \left(E_{lj}^{mn}\right),\;\mathrm{and}\;\Gamma \left(E_{lj}^{mn},E_{mn}^{lj}\right)+\Gamma^{*}\left(E_{lj}^{mn},E_{mn}^{lj}\right)\to 2\pi T\delta \left(E_{lj}^{mn}\right).$$

**Remark** **1.** *In continuous models without potential, the large-box limit is taken before the weak nonlinearity limit. This makes the momentum space continuous, and it is only for continuous space that the Dirac delta function can be introduced. In our discrete NLS, the "box" is a large lattice, but the linear modes are still discrete. However, the Dirac delta function in the above expressions is well defined because of the averaging operator, which can be viewed as an integral over the possible realisations of $E_{j}$ which form a continuous set (since the set of possible values of potentials is continuous).*

**Remark** **2.** *In continuous NLS without potential, the dispersion relation does not allow four-wave resonances in 1D. Thus, we can expect that the above expressions will become null if we make the lattice spacing in our discrete system or the maximum potential ω too small. In this case, we expect the six-wave process to be more effective than the four-wave process (see more about this later in this paper).*

Let us denote

(22) $$K\left(l-j,m-j,n-j\right)\;:=\;4\pi \epsilon^{2}\langle |V_{lj}^{mn}{|}^{2}\delta \left(E_{lj}^{mn}\right)\rangle ,$$

where we took into account the fact that all the lattice sites are equivalent and, therefore, *K* may depend of the difference of the site indices only.

Substituting (19) and (20) into (16) and taking into account (21) and that $\left(N_{j}\left(T\right)-N_{j}\left(0\right)\right)/T\approx {\dot{N}}_{j}\left(T\right)$, we have the following kinetic equation,

(23) $$\begin{array}{cc} {\dot{N}}_{j}\;= & \;\sum_{m,n,l\in Z}^{\prime }K\left(l-j,m-j,n-j\right)\left(N_{l}N_{m}N_{n}+N_{n}N_{m}N_{j}-N_{j}N_{n}N_{l}-N_{l}N_{j}N_{m}\right), \end{array}$$

This kinetic equation has a form similar to the four-wave turbulence kinetic equation [20,21] and the quantum Boltzmann equation (cf., [20,21,24,27,28,33]). In particular, all these systems conserve the total mass, $\sum_{j}N_{j}$ and have the characteristic evolution time scales as $1/\epsilon^{2}$. However, there are important differences. Firstly, because the modes are localised rather than being monochromatic waves as in usual wave turbulence, there is no conservation of momentum. Secondly, also due to the localisation, the modes are parametrised by their sites at the lattice rather than by their momenta, as in usual wave turbulence. In fact, the linear modes in our case are equivalent to each other in terms of their momentum content. Thirdly, for strong fluctuating potentials with amplitudes ω∼1, the eigenfunctions $\psi_{j}$ are strongly localised around the respective *j*. This means that in this case, K(l−j,m−j,n−j) is strongly peak near l=m=n=j and we can not to take a continuous limit in the kinetic equation.

On the other hand, the localisation is less strong for weaker ω, and the width of K(l−j,m−j,n−j) is greater. In the case when such a width is significantly greater than the lattice spacing, one can pass to the continuous limit and

(24) $$\begin{array}{c} {\dot{N}}_{j}\;=\underset{R^{3}}{\;\int \int \int \;}K\left(l-j,m-j,n-j\right)N_{j}N_{l}N_{m}N_{n}\left(N_{j}^{-1}+N_{l}^{-1}-N_{m}^{-1}-N_{n}^{-1}\right)dmdndl, \end{array}$$

## 3. The Porous Medium Equation

Let us now exploit the property of locality of the kernel in the kinetic equation arising from the localisation of the linear eigenmodes. This will allow us to reduce the integro-differential kinetic equation to a simpler nonlinear diffusion equation. This will also allow us to obtain solutions corresponding to delocalisation.

### 3.1. Derivation of the Porous Medium Equation

Let us now consider the case where the modes are not too localised so that the continuous kinetic equation works, but at the same time, the width of *K* is much less than the characteristic length of dependence of $N_{j}$ on *j*. Let us multiply equation (24) by an arbitrary function f(j) and integrate over *j*. Split the result in four equal parts and the last three parts change variables as, respectively: j↔l,m↔n; j↔m,l↔n; j↔n,l↔m. These transformations leave function *K* unchanged, so we have:

(25) $$\begin{array}{c} \int {\dot{N}}_{j}f_{j}dj\;=\frac{1}{4}\;\int \int \int \int \;K\left(l-j,m-j,n-j\right)N_{l}N_{m}N_{n}N_{j}\times \\ \left(N_{j}^{-1}+N_{l}^{-1}-N_{m}^{-1}-N_{n}^{-1}\right)\left(f_{j}+f_{l}-f_{m}-f_{n}\right)dmdndldj. \end{array}$$

Taylor expanding the expressions in both brackets to the leading order in small $\tilde{l}=l-j$, $\tilde{m}=m-j$, $\tilde{n}=n-j$, and writing $N_{l}N_{m}N_{n}N_{j}\approx N_{j}^{4}$ we have:

(26) $$\begin{array}{c} \int {\dot{N}}_{j}f_{j}dj\;=\frac{1}{4}\;\int \int \int \int \;K\left(\tilde{l},\tilde{m},\tilde{n}\right)N_{j}^{4}{\left(\tilde{l}-\tilde{m}-\tilde{n}\right)}^{2}{\left(N_{j}^{-1}\right)}^{\prime }f_{j}^{\prime }d\tilde{m}d\tilde{n}d\tilde{l}dj, \end{array}$$

where prime denotes differentiation with respect to *j*. Integrating by parts with respect to *j* and rearranging, we get:

(27) $$\begin{array}{c} \int {\dot{N}}_{j}f_{j}dj\;=-\frac{1}{4}\;\int \int \int \int \;K\left(\tilde{l},\tilde{m},\tilde{n}\right){\left(\tilde{l}-\tilde{m}-\tilde{n}\right)}^{2}\partial_{j}\left(N_{j}^{4}{\left(N_{j}^{-1}\right)}^{\prime }\right)f_{j}d\tilde{m}d\tilde{n}d\tilde{l}dj. \end{array}$$

Since $f_{j}$ is arbitrary, we can drop *j*-integration on both sides. Rearranging, we finally obtain:

(28) $${\dot{N}}_{j}=D\partial_{jj}N_{j}^{3},$$

where

(29) $$D=\frac{1}{12}\;\int \int \int \;K\left(\tilde{l},\tilde{m},\tilde{n}\right){\left(\tilde{l}-\tilde{m}-\tilde{n}\right)}^{2}d\tilde{m}d\tilde{n}d\tilde{l}.$$

Equation (28) is a nonlinear diffusion equation (with the diffusion coefficient $3DN_{j}^{2}$) which belongs to the class of porous medium equations [34]. Let us consider now an extension of (28):

(30) $$\partial_{t}N\left(t,k\right)=\partial_{kk}N^{m}\left(t,k\right),\;\;\;m>1,$$

where we keep in mind that m=3 and m=5 correspond to the four-wave and the six-wave systems, respectively. Later, we will discuss the conditions under which the six-wave dynamics occurs Let us now consider solutions of this equation porous medium equation.

### 3.2. Steady State Solutions-Ohm’s Law

The porous medium equation has a conservation law form, so we will use here an electricity terminology, i.e., we will call *N* a charge density. The steady state solution of the porous medium equation takes the form

(31) $$\partial_{kk}N^{m}\left(k\right)\;=\;0.$$

As a consequence

(32) $$\partial_{k}N^{m}\left(k\right)\;=\;-J,$$

where *J* is a current constant. Integrating once more, we get $N^{m}\left(k\right)\;=\;A-Jk,$ where *A* is a constant, and hence

(33) $$N\left(k\right)\;=\;{\left[A-Jk\right]}^{\frac{1}{m}}.$$

As we see, for A,J>0 the charge density drops to zero at a location k=a=A/J. Since *J* is a *k*-independent constant, one must put an "electrode" at k=a that absorbs current *J*.

Let us now introduce a potential ϕ via

(34) $$\phi^{\prime \prime }\;=\;N,$$

which can be solved directly,

(35) $$\phi \;=\;\frac{m^{2}}{\left(2m+1\right)\left(m+1\right)J^{2}}{\left[A-Jk\right]}^{\frac{1}{m}+2}\;+\;Dk\;+\;P.$$

where *D* and *P* are some constants. Since the potential is defined up to a constant only, we can fix it by condition ϕ(a)=0, which gives Da+P=0. In other words, the electrode at *a* is a “ground”. Also, *D* has a meaning of a constant part of the electric field. It is actually not observable in our problem, so we set D=P=0 leading to A=Ja.

For "voltage", we find

(36) $$\begin{array}{c} V=\phi \left(0\right)-\phi \left(a\right)\;=\frac{m^{2}}{\left(2m+1\right)\left(m+1\right)J^{2}}A^{\frac{1}{m}+2}=\frac{m^{2}a^{\frac{1}{m}+2}}{\left(2m+1)(m+1\right)}J^{\frac{1}{m}}. \end{array}$$

This is an analog of Ohm’s law describing the relation between the voltage and current. Note that in our case, the remaining traces of the localisation effect lead to nonlinearity of Ohm’s law. However, since the current *J* is finite for any V>0, the medium is conducting, i.e., the localisation (insulator) property is broken by nonlinearity.

### 3.3. Self-Similar Solutions

Let us now consider another, more traditional approach to examining delocalisation; time dependent evolution (spreading) of initially localised distributions. Essentially, most of the results of this sections were previously obtained in [5,35,36], and here, we reproduce and summarise them for completeness of discussion.

Let us look for self-similar solutions of the first kind of the equation of (28),

(37) $$N\left(t,k\right)\;=\;t^{b}f\left(\xi \right),$$

where $\xi =kt^{a},\;\;k,t>0$, and *a* and *b* are some constants. For consistency of the formulation, we must satisfy 2a=−1+b(1−m). The total mass conservation, ∫N(t,k)dk=const, gives the second condition: a=b. Thus,

(38) $$\xi \;=\;kt^{-\frac{1}{\left(m+1\right)}}.$$

The rate of spreading of initially localised distributions is usually measured by the evolution of the variance defined as $\sigma =\int k^{2}N\left(k,t\right)dk.$ In our case we have

(39) $$\sigma \left(t\right)=\int k^{2}t^{-\frac{1}{\left(m+1\right)}}f\left(kt^{-\frac{1}{\left(m+1\right)}}\right)dk=t^{\frac{2}{\left(m+1\right)}}\int \xi^{2}f\left(\xi \right)d\xi ,$$

so $\sigma \left(t\right)∼t^{\frac{2}{\left(m+1\right)}}$ which is usually refered to as a sub-diffusive spreading. In particular, for the four-wave systems we have $\sigma \left(t\right)∼t^{1/2}$ and for the six-wave systems, respectively, $\sigma \left(t\right)∼t^{1/3}$.

Plugging (37) into (30) yields the following equation for *f*,

(40) $$\left(m+1\right){\left(f^{m}\right)}^{\prime \prime }\;+\;\xi f^{\prime }\;+f=0.$$

Integrating this equation once, we get

(41) $$\left(m+1\right){\left(f^{m}\right)}^{\prime }\;+\;\xi f=C,$$

where C=const. Let us consider first the case with C≠0: according to the above equation ${\left(f^{m}\right)}^{\prime }<C/\left(m+1\right)$, so the solution has a sharp front at $\xi =\xi^{*}$ such that $f(\xi^{*})=0$. (This front will be on the right boundary of the solution for C>0 and on the left boundary for C<0.) But the current $J=-\partial_{k}N^{m}=t^{-1}{\left(f^{m}\right)}^{\prime }$ will remain finite at $\xi =\xi^{*}$, which means that there is a moving sink of particles at $k=\xi^{*}t^{-a}$. Thus, the solutions with C≠0 are unphysical.

Thus, we put C=0 and solve equation (41) directly, which gives [36]

(42) $$f\left(\xi \right)\;=\;{\left[\frac{\left(m-1\right)}{2m(m+1)}\left(\xi^{*2}-\xi^{2}\right)\right]}^{\frac{1}{m-1}}$$

with some constant $\xi^{*}$ which, again, corresponds to a sharp moving boundary of the solution. Considering a negative *k*, one can see that the solution remains in the same form (42). Thus, including both negative and positive *k*, we have a solution with the shape of a droplet with sharp boundaries expanding on a flat surface.

This shows that one can construct a solution to the porous medium equation which spreads for large times. Moreover, the theory of porous medium equations says that such a self-similar solution is stable, and that it is an attractor for all solutions with arbitrary localised initial conditions. This result applies to any m>1, including m=3 (four-wave regime considered in this paper) and m=5 (six-wave regime outlined in the next section). Therefore this answers the “weak turbulence” question (3) positively.

## 4. Six-Wave Regime

Here, we will present a speculative discussion of the cases when the four-wave interaction considered in this paper may become ineffective and subdominant to a higher-order process, the six-wave regime. Let ω (the maximal strength of the potentials) be small compered to $k^{2}$ (*k* being the typical wave momentum), and k≪1. In the limit, we get the continuous NLS without potentials, which is integrable and, therefore, the resonant interactions of every order are null. Small deviation from the limit of zero potentials and continuous space result in small nonintegrability and, therefore, in activation of wave resonances. However, the four-wave process is still null, because the four-wave frequency and momentum resonant conditions cannot be satisfied in 1D for the dispersion relations $E=k^{2}$, and this property cannot be removed by small perturbations. Due to the U(1) symmetry of the problem, the odd-order resonant processes are absent, and the leading order process is expected to be six-wave.

Based on analogy with equation (24), we can conjecture that the six-wave kinetic equation in this case will take the form

(43) $$\begin{array}{c} {\dot{N}}_{j}\;=\underset{R^{3}}{\;\int \int \int \;}L\left(l-j,m-j,n-j,p-j,q-j\right)N_{j}N_{l}N_{m}N_{n}N_{p}N_{q}\\ (N_{j}^{-1}+N_{l}^{-1}+N_{m}^{-1}-N_{n}^{-1}-N_{p}^{-1}-N_{q}^{-1})dmdndldpdq, \end{array}$$

where

(44) $$L\left(l-j,m-j,n-j,p-j,q-j\right)\;:=\;\epsilon^{4}\langle |W_{jlm}^{npq}{|}^{2}\delta \left(E_{jlm}^{npq}\right)\rangle$$

with $W_{jlm}^{npq}$ being an interaction coefficient which is probably quite complicated, since obtaining it should involve a canonical transformation removing the cubic nonlinearity from the dynamical equation (as it is usual when the four-wave process is absent). It is natural to expect that in this case, the energy and momentum will be approximately conserved and the kernel *L* will be weakly localised near values j∼l∼m∼n∼p∼q. The closer we are to the limit of the zero potentials and continuous medium, the better the energy/momentum conservation, and the weaker the localisation in the physical space.

## 5. Summary and Discussion

In the present paper, we developed a wave turbulence theory for the description of weak excitations in the model described by the discrete one-dimensional NLS equation with random potentials (2). We systematically derived a four-wave kinetic equation (23) and its continuous version (24). From the latter, we derived a porous medium equation (28) for the cases when the linear mode localisation length is less than the characteristic length of the wave spectrum variation. Such a porous medium equation was previously suggested for the discrete NLS model in [5,35], and in the present paper, we elevate the status of this equation as fully justified via a systematic wave turbulence derivation. Further, we presented a speculative argument about the conditions when the four-wave regime is replaced by a six-wave process described by the kinetic Equation (43) and (in the case of slow spatial variations of the wave spectra) by the m=5 version of the porous medium Equation (30).

Analysing stationary solutions of the porous medium equation, we have obtained an effective Ohm’s law; a nonlinear current-voltage relation indicating that weak nonlinearity makes the lattice a conductor. This is one of the ways to the localisation.

Another more traditional way to characterise de-localisation by nonlinearity is to study the self-similar solution of the porous medium Equation (30). For any m>1, the self-similar spreading appears to be sub-diffusive: regime $\sigma \left(t\right)∼t^{1/2}$ is realised for the four-wave case (m=2) whereas the six-wave regime (m=3) leads to $\sigma \left(t\right)∼t^{1/3}$. Numerical experiments of [35] reported observation of the $\sigma \left(t\right)∼t^{1/2}$ regime, whereas other numerical experiments [5,6,9,10] reported $\sigma \left(t\right)∼t^{1/2}$. Interestingly, a $\sigma \left(t\right)∼t^{1/3}$ to $\sigma \left(t\right)∼t^{1/2}$ was observed in [5,6,10] when the wave phases where artificially scrambled. A connection between the degree of phase randomness and realisability of either four-wave or six-wave dynamics remains to be understood. It is quite possible that the phases become random only after passing to variables obtained via the canonical transformation (required for deriving the six-wave kinetic equation) and that the phases of the original variables are correlated.

The extensive and expansive numerics on the original equation were done in [5,6,10], in which they get the 1/3 growth, pointing to the six-wave equation discussed in our work. Moreover, with extra dephasing, the growth exponent was shown to be 1/2, which corresponds to the four-wave process. Doing numerics in order to verify our assumptions is beyond the scope of this work and will be the subject of a different paper. The main thrust of this work is to show that a new approach is available to study the fundamental problem of chaotic behavior driven by nonlinearity in a dispersive hamiltonian system like NLS or FPUT models. The second main point is that this model has physically meaningful randomness, that may be relevant to the derivation and emergence of the wave turbulent behavior. In this work, we do not claim a rigorous proof of this kinetic equation or dispersion; that would be indeed a more mathematically oriented paper. Instead, we have identified in this paper, explicit conjectures, mostly about the behavior of the linear system with random potential, that leads to the above kinetic equations and resulting dispersion. 
